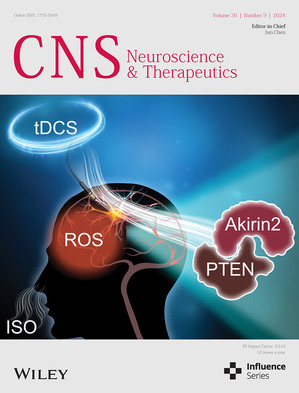# Additional Cover

**DOI:** 10.1111/cns.70074

**Published:** 2024-09-26

## Abstract

The cover image is based on the article *Transcranial direct current stimulation enhances the protective effect of isoflurane preconditioning on cerebral ischemia/reperfusion injury: A new mechanism associated with the nuclear protein Akirin2* by Xiangyi Kong et al., https://doi.org/10.1111/cns.70033.